# Defect-Driven and Age-Related Patterns of Flap Selection in Pediatric Mandibular Reconstruction

**DOI:** 10.3390/jcm15114244

**Published:** 2026-05-30

**Authors:** Dominika Lech, Robert Maksymowicz, Jeremi Matysek, Cyprian Strączek, Aleksandra Strzelecka, Marcin Kozakiewicz, Łukasz Krakowczyk, Krzysztof Dowgierd

**Affiliations:** 1 Department of Clinical Pediatrics, Head and Neck Surgery Clinic for Children and Young Adults, University of Warmia and Mazury, 10-709 Olsztyn, Poland; 2 Department of Maxillofacial Surgery, Medical University of Lodz, 113 Żeromskiego Str., 90-549 Lodz, Poland; 3 2nd Department of Oncologic Surgery, Maria Sklodowska Curie Memorial National Cancer Center, 44-100 Gliwice, Poland

**Keywords:** pediatric mandibular reconstruction, free flap, donor-site selection, Brown classification, mandibular defects

## Abstract

**Background:** Pediatric mandibular reconstruction requires restoration of mandibular continuity while accounting for craniofacial growth, dental development, and long-term functional outcomes. Although vascularized osseous free flaps are widely used, the influence of defect characteristics and patient age on donor-site selection remains unclear. This study evaluated patterns of flap selection in relation to mandibular defect classification and patient age. **Methods:** This retrospective single-center cohort study included 75 patients aged 1–18 years who underwent microvascular mandibular reconstruction between 2011 and 2025. Donor sites included the fibula, iliac crest, and medial femoral condyle. Mandibular defects were classified according to the Brown classification. Associations between defect class, age, donor-site selection, and flap loss were analyzed. **Results:** The fibula flap was used most frequently (52.0%), followed by the iliac crest (44.0%) and medial femoral condyle (4.0%). Donor-site selection was significantly associated with Brown defect classification (*p* < 0.001). Age differed across donor-site groups, with fibula flaps used more often in younger patients and iliac crest flaps in older patients. However, after stratification by defect class, age-related differences were not statistically significant. Overall flap survival was 93.3%, and age was not associated with total flap loss. **Conclusions:** Donor-site selection showed a stronger association with defect characteristics than with patient age. Observed age-related patterns likely reflect differences in defect morphology. These findings support the clinical relevance of a defect-oriented framework for pediatric mandibular reconstruction; however, flap selection remains individualized and influenced by multiple patient- and surgeon-related factors.

## 1. Introduction

Pediatric mandibular defects present a unique reconstructive challenge, requiring restoration of a load-bearing osseous structure essential for occlusion, mastication, lower facial contour, and craniofacial symmetry. Unlike midfacial reconstruction, which often involves complex three-dimensional restoration and separation of anatomical compartments, mandibular reconstruction places particular emphasis on re-establishing bony continuity. In children and adolescents, this process is further complicated by ongoing craniofacial growth, dental eruption, and long-term adaptation of reconstructed tissues within the developing facial skeleton. Consequently, treatment strategies must address not only immediate function but also future craniofacial development [1,2,3,4,5].

Microvascular reconstruction using vascularized bone transfer has become the standard approach for managing segmental mandibular defects. Since the introduction of the fibula free flap by Hidalgo, these techniques have enabled reliable restoration of mandibular form and function with excellent outcomes [6]. The fibula free flap remains the mainstay of treatment due to its consistent vascular anatomy, adequate bone length, and versatility in reconstructing composite defects [4,5,6,7]. However, alternative donor sites, including the deep circumflex iliac artery and medial femoral condyle flaps, may offer specific structural and contour advantages in selected clinical scenarios [2,7,8,9,10,11].

Flap selection in mandibular reconstruction is primarily determined by defect characteristics, including location, extent, and involvement of key anatomical structures [2,7,12]. Classification systems, including the Brown mandibular defect classification, provide a structured framework for describing defect extent and guiding reconstructive decision-making [13]. Accordingly, donor sites are not interchangeable but are selected based on specific reconstructive requirements. However, despite the recognized importance of defect characteristics in surgical planning, the relationship between standardized mandibular defect classification and donor-site selection has not been systematically evaluated in pediatric populations [14,15,16]. Moreover, it remains unclear whether observed differences in flap choice across age groups reflect age-specific surgical preferences, differences in defect morphology, or both.

Although surgical planning in children must account for growth, dental development, donor-site morbidity, and long-term craniofacial adaptation, flap selection is also strongly influenced by the anatomical characteristics of the mandibular defect [2,7,12]. In clinical practice, patient age may affect reconstructive preferences, particularly in relation to bone size, expected growth, dentition, and the technical feasibility of specific donor sites. However, age-related differences in flap selection may also reflect differences in defect extent, location, and reconstructive requirements [4,7,17,18].

To address this issue, the present study analyzes a single-center cohort of pediatric patients undergoing microvascular reconstruction of mandibular defects. The primary aim was to describe donor-site selection patterns in relation to defect characteristics, with particular reference to the Brown mandibular defect classification. A secondary aim was to evaluate age-related patterns in flap choice and to assess whether patient age was associated with total flap loss. By examining both defect-related and age-related patterns, this study seeks to provide a more structured description of reconstructive decision-making in pediatric mandibular reconstruction.

## 2. Materials and Methods

### 2.1. Study Design and Ethical Approval

This retrospective cohort study included pediatric patients who underwent microvascular free-flap reconstruction of the mandible between August 2011 and September 2025 at the Division of Maxillofacial Surgery for Children and Young Adults, Head and Neck Clinic, Regional Specialized Children’s Hospital in Olsztyn, Poland.

### 2.2. Patient Selection

Eligible patients were aged 1–18 years at the time of surgery and underwent mandibular reconstruction using a microvascular free flap. Three types of osseous free flaps (fibula, iliac crest, and medial femoral condyle) were included. Cases were identified from institutional surgical records. Only patients with complete electronic medical documentation were eligible for inclusion. Patients with incomplete records were excluded.

A total of 75 consecutive patients met the inclusion criteria and were included in the final analysis.

### 2.3. Data Collection

Clinical data were extracted retrospectively from electronic health records, including operative reports, pathology reports, and inpatient documentation. The following variables were recorded:

- Age at the time of surgery;
- Sex;
- Etiology of defect;
- Extent of mandibular defect according to the Brown classification;
- Donor site;
- Occurrence of total flap loss.

Tumor type, where applicable, was recorded for descriptive characterization of the cohort. Total flap loss was defined as complete flap necrosis requiring surgical removal of the flap. All data were anonymized prior to analysis.

### 2.4. Age Categorization

Age was analyzed as a continuous variable. In addition, patients were categorized into four predefined age groups:

- ≤5 years;
- 6–10 years;
- 11–15 years;
- >15 years.

### 2.5. Surgical Decision-Making

Flap selection was determined by the operating surgical team based on patient-specific and defect-specific factors within a multidisciplinary clinical setting. Although no formalized protocol was applied, flap choice reflected general reconstructive principles used in the institution, including defect extent, reconstructive requirements, and anticipated functional outcomes. Patient age was considered during surgical planning, but did not represent an isolated determinant of flap selection. Therefore, the present analysis should be interpreted as an evaluation of observed associations between clinical variables and donor-site selection rather than as a reconstruction of a standardized decision-making algorithm.

All procedures were performed using a two-team approach, with one team responsible for tumor resection or defect preparation and the second team performing flap harvest, inset, and microvascular anastomosis.

### 2.6. Statistical Analysis

Statistical analysis was performed using STATGRAPHICS Centurion 19 (StatPoint Technologies, Tulsa, OK, USA). Continuous variables are presented as mean ± standard deviation (SD) and median values. Categorical variables are presented as frequencies and percentages. Because subgroup sizes were unequal and distributions were not assumed to be normal, nonparametric methods were primarily used for comparisons involving age. Differences in age between donor-site groups were assessed using the Kruskal–Wallis test, followed by Dunn’s post hoc test where appropriate. Additional pairwise comparisons between iliac crest and fibula groups were performed using the Mann–Whitney U test. Differences in age according to flap survival were assessed using the Mann–Whitney U test. After excluding patients with non-oncological etiology, differences in age across Brown defect classes were assessed using the Kruskal–Wallis test, followed by Dunn’s post hoc test for pairwise comparisons where appropriate. Associations between categorical variables, including Brown classification and donor-site selection, were evaluated using the chi-square test of independence. When expected cell counts were low, results were interpreted with caution. The association between age group and donor-site selection was further assessed using a linear-by-linear association test and Spearman’s rank correlation coefficient. Firth penalized logistic regression was used to evaluate age as a potential predictor of total flap loss due to the low number of events. Multivariable modelling of donor-site selection was not performed because of limited sample size and sparse subgroup distribution, which would have resulted in unstable estimates and a high risk of overfitting. A two-sided *p*-value < 0.05 was considered statistically significant.

## 3. Results

The study cohort consisted of 75 pediatric patients who underwent microsurgical free-flap reconstruction of mandibular defects. There were 44 males (58.67%) and 31 females (41.33%). The largest age subgroup comprised patients aged 11–15 years (n = 34; 45.33%), followed by those older than 15 years (n = 21; 28.00%), those aged 6–10 years (n = 15; 20.00%), and those aged 5 years or younger (n = 5; 6.67%).

Oncological defects were the most common indication for reconstruction (n = 59; 78.67%), followed by congenital defects (n = 14; 18.67%) and iatrogenic defects (n = 2; 2.67%). The fibula was the most frequently used donor site (n = 39; 52.00%), followed by the iliac crest (n = 33; 44.00%) and the medial femoral condyle (n = 3; 4.00%).

Overall flap survival was 93.33% (70/75), while total flap loss occurred in 5 patients (6.67%). Detailed demographic and clinical characteristics are presented in Table 1.

### 3.1. Age and Donor-Site Selection in Mandibular Reconstruction

Table 2 summarizes patient age according to donor site. The number of reconstructions performed with the iliac crest and fibula was comparable (n = 33 vs. n = 39). Patients reconstructed with the iliac crest were older, with a mean age of 13.79 ± 2.96 years and a median age of 14.00 years, whereas those reconstructed with the fibula had a mean age of 11.44 ± 4.59 years and a median age of 13.00 years. Three patients underwent reconstruction with a medial femoral condyle flap; their mean and median age was 16.00 years. 

The Kruskal–Wallis test showed a significant difference in age distribution across donor-site groups (H(2) = 6.55, *p* < 0.05).

**Figure 1:**
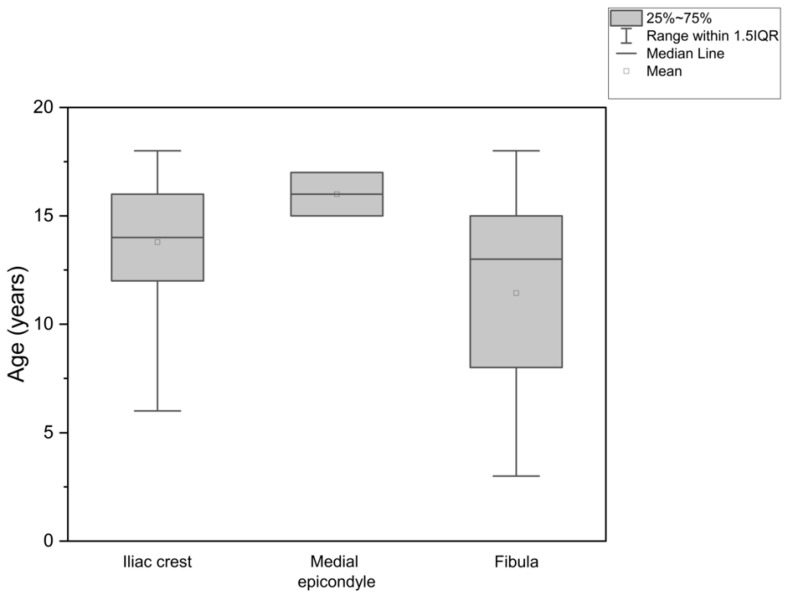
Box plot showing patients’ age distribution at each donor site.

Post hoc analysis with Dunn’s test did not reveal significant pairwise differences between donor-site groups. Because the medial femoral condyle group was very small (n = 3) and clinically distinct, an additional Mann–Whitney test was performed to compare the iliac crest and fibula groups. This analysis demonstrated a significant difference in age between these two groups (U = 465, *p* < 0.05). The corresponding age distribution is shown in Figure 1.

### 3.2. Age, Brown Classification, and Donor-Site Selection

Table 3 summarizes patient age according to mandibular defect extent as defined by the Brown classification. The largest subgroup comprised patients with class II defects (hemimandibulectomy with ipsilateral canine) (n = 42). Mean age ranged from 12.25 ± 6.50 years in class I defects to 17.00 ± 1.41 years in patients with class IIc defects.

After excluding patients with non-oncological etiology from the analysis, the relationship between patient age and Brown classification was assessed using the non-parametric Kruskal–Wallis test. No statistically significant differences in age distribution were observed across Brown classes, H(5) = 5.63, *p* = 0.344. Dunn’s post hoc test likewise showed no significant pairwise differences between groups. These findings indicate that patient age did not differ significantly across mandibular defect classes as defined by the Brown classification. Although patients with class IIc and IVc defects tended to be older on descriptive analysis, these differences did not reach statistical significance.

The relationship between the type of mandibular defect according to the Brown classification and the donor site used for reconstruction was evaluated using a chi-square test of independence. In the study of 75 patients, the fibula flap was the most frequently used (n = 39; 52.0%), followed by the iliac crest (n = 33; 44.0%) and the medial femoral condyle (n = 3; 4.0%).

Class II defects constituted the largest subgroup (n = 42; 56.0% of the cohort). Within this group, the iliac crest was used in 26 cases (34.7% of the entire cohort), whereas the fibula was used in 16 cases (21.3%). In class Ic defects (n = 5), the iliac crest was used in three patients and the fibula in two patients. In class I defects (n = 4), reconstruction was performed equally with the iliac crest and fibula (two cases each). For class IIc defects (n = 2), one case was reconstructed using the iliac crest and one using the fibula. Class III defects (n = 4) showed a more heterogeneous distribution: the medial femoral condyle flap was used in two cases, while the iliac crest and fibula were each used in one case. Extensive class IVc defects (n = 2) were reconstructed exclusively using the fibula.

The chi-square test of independence demonstrated a statistically significant association between the type of mandibular defect and the donor site used for reconstruction (χ^2^(10) = 31.79, *p* = 0.0004), indicating that the choice of donor site was significantly related to the extent of the mandibular defect.

### 3.3. Age-Related Donor-Site Selection Within Brown Defect Classes

To further explore whether age-related differences in donor-site selection persisted within specific mandibular defect categories, patients were stratified according to the Brown classification. Comparisons were limited to Brown classes in which both iliac crest and fibula flaps were used.

**Figure 2:**
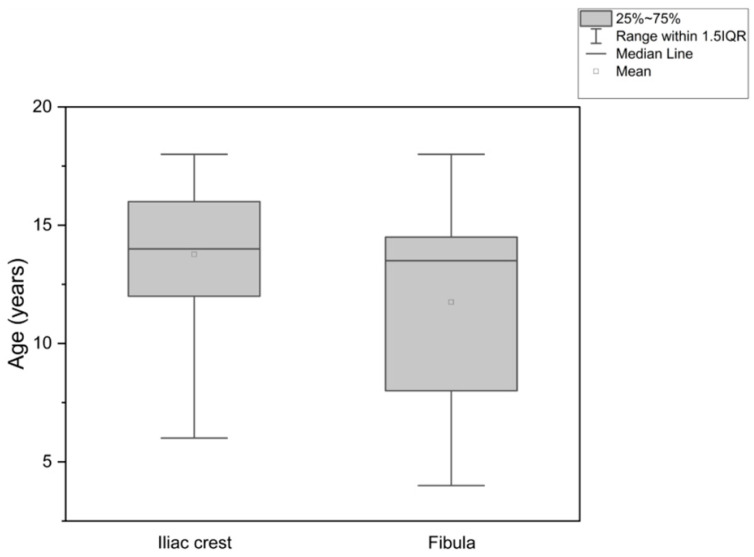
Age distribution according to donor site in patients with Brown class II mandibular defects.

In class I defects, no significant difference in age was observed between patients reconstructed with iliac crest and fibula flaps (median age: 14.0 vs. 10.5 years; U = 2.00, *p* = 1.00). Similarly, no significant age difference was found in class Ic defects (median age: 10.0 vs. 14.5 years; U = 4.00, *p* = 0.77). In class II defects, which represented the largest subgroup, patients reconstructed with iliac crest flaps were older on descriptive analysis than those reconstructed with fibula flaps; however, this difference did not reach statistical significance (mean age: 13.77 ± 2.82 vs. 11.75 ± 4.07 years; median age: 14.0 vs. 13.5 years; U = 152.5, *p* = 0.15; Figure 2).

Statistical comparisons were not performed for class IIc, III, and IVc defects because of very small subgroup sizes and/or the presence of only one donor-site category. Overall, after stratification by Brown defect class, age-related differences in donor-site selection were not statistically significant within individual defect categories.

### 3.4. Age and Flap Survival

Table 4 summarizes patient age according to flap survival status. The mean age was 12.84 ± 4.02 years in patients with flap survival and 10.00 ± 4.06 years in those with total flap loss.

**Figure 3:**
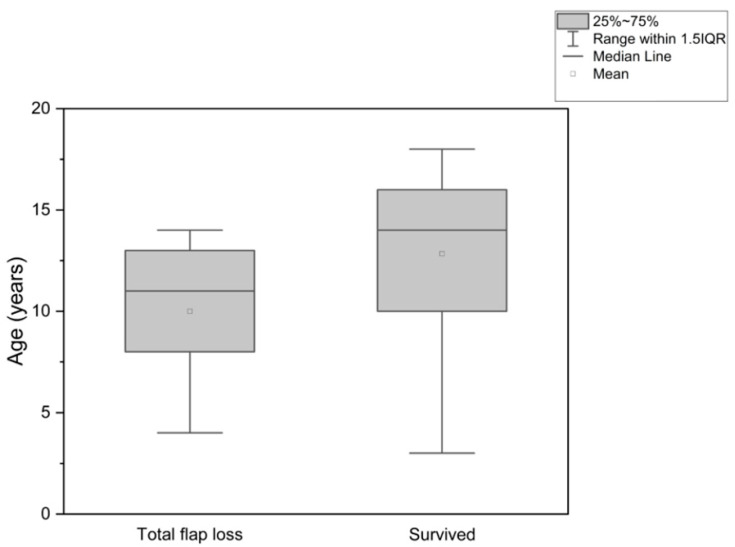
Box plot showing patient age distribution according to flap survival status.

A Mann–Whitney test was performed to compare the age between patients with flap survival and those with total flap loss. As shown in Figure 3, no statistically significant difference was observed between the groups (U = 97.5, *p* = 0.10).

To further evaluate the relationship between age and the risk of total flap loss, a Firth penalized logistic regression analysis was performed. Patient age was not a significant predictor of total flap loss (odds ratio [OR] = 0.86, 95% CI: 0.71–1.05, *p* = 0.14).

Although patients with flap loss tended to be younger on descriptive analysis, this trend did not reach statistical significance.

Overall, these findings indicate that patient age was not associated with the occurrence of total flap loss in this cohort.

### 3.5. Age Group and Donor-Site Selection

Table 5 shows the distribution of donor sites across four age groups. In patients aged 5 years or younger, all reconstructions were performed using the fibula flap. In the 6–10-year group, the fibula remained the most commonly used donor site. In contrast, among patients aged 11–15 years and those older than 15 years, the iliac crest was used more frequently. Medial femoral condyle flaps were used only in patients older than 11 years.

A linear association test demonstrated a significant association between age group and donor-site selection (χ^2^ = 5.79, df = 1, *p* = 0.016). Spearman’s rank correlation analysis showed a significant negative correlation between age group and donor-site category (ρ = −0.25, n = 75, *p* < 0.05), indicating an age-related shift in donor-site selection across the cohort.

## 4. Discussion

The present study shows that donor-site selection in pediatric mandibular microvascular reconstruction was associated with mandibular defect extent, as defined by the Brown classification. Age-related differences in flap choice were observed in the overall cohort, particularly with more frequent fibula use in younger patients and more frequent iliac crest use in older children and adolescents. However, these age-related differences were not statistically significant after stratification by Brown defect class. Patient age was also not significantly associated with total flap loss.

This relationship can be explained by the underlying reconstructive requirements. The Brown classification provides a structured description of defect location and extent, which directly translates into specific biomechanical and functional needs, including restoration of mandibular continuity, adequate bone length, vertical height, and contour [13]. In more extensive defects, particularly those involving multiple segments or the condyle, reconstruction must address not only structural stability but also occlusion and temporomandibular joint function. Consequently, flap selection is primarily dictated by these anatomical and functional demands rather than patient-related factors alone. This concept is consistent with previous studies demonstrating that reconstructive strategies in pediatric mandibular defects are largely determined by defect size and characteristics, with larger defects requiring vascularized bone reconstruction, whereas smaller defects may be managed with nonvascularized grafts [1,19]. Although classification systems such as the Brown classification provide a valuable framework for surgical planning, they should not be interpreted as rigid algorithms, particularly in pediatric populations, where growth potential and long-term functional considerations necessitate an individualized approach [13].

An association between patient age and donor-site selection was observed at the cohort level, with younger patients more frequently undergoing fibula-based reconstruction and older patients more often reconstructed using iliac crest flaps. This pattern may reflect several practical and biological factors, including increasing bone volume and donor-site dimensions with age, the need for greater vertical bone height in the context of dental rehabilitation and implant planning, as well as differences in donor-site morbidity and technical feasibility across age groups. In pediatric patients, reconstructive planning is further complicated by the fact that both donor and recipient sites remain actively involved in craniofacial growth and development, necessitating an individualized approach to surgical decision-making [7,17]. Although growth-related factors may influence reconstructive strategy, their effects are complex and not fully predictable, as postoperative mandibular development depends on multiple variables beyond age alone, including condylar preservation and adjuvant therapy [18,20]. Importantly, after stratification according to defect extent using the Brown classification, the effect of age was no longer statistically significant. This finding suggests that the observed age-related patterns in flap selection are likely secondary to underlying differences in defect characteristics rather than representing an independent determinant of reconstructive decision-making.

In our cohort, the distribution of donor-site selection appears consistent with the inherent properties of each flap. The fibula free flap remains the most versatile option, particularly for segmental defects, as it provides long vascularized bone segments, allows multiple osteotomies, and enables reliable restoration of mandibular continuity [4,5,6,21]. The iliac crest flap offers specific advantages in selected cases, including greater vertical bone height and a favorable contour, which may be beneficial when increased bone volume is required, especially for future implant-based rehabilitation [9,11]. The medial femoral condyle flap, although used less frequently in our series, may be considered for smaller or moderately sized defects requiring precise contouring rather than extensive structural reconstruction [2,22]. This distribution is consistent with the reconstructive requirements associated with different defect types.

The flap survival rate observed in the present cohort (93.33%) is consistent with findings from recent literature, including a systematic review and meta-analysis by Huang et al., which reported a pooled survival rate of approximately 96% in pediatric mandibular reconstruction. Reported infection rates also remain relatively low (around 9%), supporting the overall safety profile of microvascular free-flap procedures in this population [16,23]. Importantly, in the present study, patient age was not significantly associated with total flap loss, suggesting that younger age alone does not represent an independent risk factor for flap failure.

Based on these findings, pediatric mandibular reconstruction may be best conceptualized within a defect-oriented, age-contextualized framework. The initial assessment should include a systematic evaluation of the defect, including Brown class, defect length, anatomical location, involvement of the condyle or anterior segment, and associated soft-tissue requirements [13]. These factors reflect the main reconstructive demands and may inform donor-site selection. This concept is consistent with pediatric reconstructive literature, in which defect size and structural requirements are considered key factors in determining the need for vascularized bone transfer [1]. Once a suitable donor site has been selected according to defect-related requirements, the reconstructive plan should be further individualized according to patient-related factors, including craniofacial growth, dental development, feasibility of future implant-based rehabilitation, donor-site morbidity, and the likelihood of staged procedures. This is particularly important in children, as both donor and recipient sites remain subject to growth and development [17,24], and postoperative mandibular growth following vascularized reconstruction is influenced by multiple factors beyond age alone, including condylar preservation and adjuvant therapy [18,25]. Therefore, age should be interpreted as one of several contextual factors considered within individualized surgical planning, rather than as an isolated determinant of flap selection.

This study has several limitations. First, its retrospective, single-center design introduces the possibility of selection bias and limits the ability to infer causal relationships. Donor-site selection was based on individualized surgeon judgment rather than a standardized, prospectively defined protocol. As a result, the observed association between Brown defect classification and donor-site selection should be interpreted as a descriptive selection pattern rather than evidence that defect classification was the definitive primary determinant of flap choice. In addition, although the Brown classification was used as a standardized measure of mandibular defect extent, it does not account for all factors relevant to donor-site selection. Second, subgroup sizes were uneven, with several Brown classification categories represented by only a small number of patients, and the medial femoral condyle group, including only three cases. This limited the robustness of subgroup comparisons and restricted the ability to draw firm conclusions regarding donor-site preferences within specific defect subtypes. In addition, the lack of statistically significant age-related differences after stratification by defect class should be interpreted cautiously, as it may reflect limited statistical power rather than the absence of a clinically relevant association. Fourth, due to the relatively small sample size and sparse distribution across subgroups, multivariable modelling was not feasible, and the independent effects of age and defect classification could not be reliably separated. In addition, the number of total flap loss events was low, which limited the statistical power to identify predictors of flap failure. Finally, this study focused on donor-site selection patterns and early surgical outcomes. Long-term outcomes, including mandibular growth, facial symmetry, occlusal development, temporomandibular joint function, dental rehabilitation, and donor-site morbidity, were not assessed and remain important areas for future investigation.

## 5. Conclusions

In this retrospective single-center pediatric cohort, donor-site selection was significantly associated with mandibular defect classification, while apparent age-related differences were attenuated after stratification by defect type. These findings indicate that defect morphology is an important factor in flap selection, with age serving as a contextual variable in surgical planning. Total flap loss was uncommon, but flap survival should be regarded as an early surgical endpoint rather than a comprehensive measure of reconstructive success. Although interpretation is limited by the retrospective design, absence of a standardized selection protocol, sparse subgroups, and lack of long-term functional, growth-related, occlusal, temporomandibular joint, and rehabilitative outcomes, the results support the clinical relevance of defect-oriented planning in pediatric mandibular reconstruction.

## Figures and Tables

**Table 1:** Demographic and clinical characteristics of the study cohort.

	Number of Patients	Percentage of the Total Number of Patients
Sex		
Female	31	41.33%
Male	44	58.67%
Age group		
≤5 years old	5	6.67%
6–10 years old	15	20.00%
11–15 years old	34	45.33%
>15 years old	21	28.00%
Etiology		
Congenital	14	18.67%
Oncological	59	78.67%
Iatrogenic	2	2.67%
Donor site		
Iliac crest (deep circumflex iliac artery flap)	33	44.00%
Medial femoral condyle	3	4.00%
Fibula	39	52.00%
Tumor type		
Not applicable	16	21.33%
Benign	51	68.00%
Malignant	8	10.67%
Brown classification group		
I	4	5.33%
Ic	5	6.67%
II	42	56.00%
IIc	2	2.67%
III	4	5.33%
IVc	2	2.67%
Total flap loss		
No	70	93.33%
Yes	5	6.67%
Total	75	100%

**Table 2:** Patient age according to donor site.

Type of Donor Site	Number of Patients	Mean Age (Standard Deviation) [Years]	Median Age [Years]
Iliac crest (deep circumflex iliac artery flap)	33	13.79 (±2.96)	14.00
Medial femoral condyle	3	16.00 (±1.00)	16.00
Fibula	39	11.44 (±4.59)	13.00
Total	75	12.65 (±4.06)	14.00

**Table 3:** Patient age according to Brown classification of mandibular defects.

Mandibular Defect According to Brown Classification	Number of Patients	Mean Age (Standard Deviation) [Years]	Median Age [Years]
I—lateral, not including canine or condyle	4	12.25 (±6.5)	14.00
Ic—lateral with condyle	5	13.00 (±4.12)	13.00
II—hemimandibulectomy with ipsilateral canine	42	13.00 (±3.45)	14.00
IIc—hemimandibulectomy with condyle	2	17.00 (±1.41)	17.00
III—anterior, including both canines	4	14.75 (±2.63)	15.5
IVc—extensive, including canines, angles, and condyles	2	16.00 (±1.41)	16.00
Not applicable	16	10.25 (±4.73)	8.5

**Table 4:** Patient age according to flap survival status.

Free Flap Status	Number of Patients	Mean Age (Standard Deviation) [Years]	Median Age [Years]
Survived	70	12.84 (±4.02)	14.00
Total flap loss	5	10.00 (±4.06)	11.00
Total	75	12.65 (±4.06)	14.00

**Table 5:** Distribution of donor sites across age groups.

Age Group	Type of Donor Site		
	Iliac Crest	Medial Femoral Condyle	Fibula
≤5 years old	0	0	5
6–10 years old	4	0	11
11–15 years old	19	1	14
>15 years old	10	2	9
Total	33	3	39

## Data Availability

The data presented in this study are available on request from the corresponding author.
